# Analysis of a Blended, In-Service, Continuing Education Course in a Public Health System: Lessons for Education Providers and Healthcare Managers

**DOI:** 10.3389/fpubh.2020.561238

**Published:** 2020-11-26

**Authors:** Fernanda Manzini, Eliana Elisabeth Diehl, Mareni Rocha Farias, Rosana Isabel dos Santos, Luciano Soares, Norberto Rech, Andrigo Antonio Lorenzoni, Silvana Nair Leite

**Affiliations:** ^1^Postgraduate Program in Pharmacy, Federal University of Santa Catarina, Florianópolis, Brazil; ^2^Department of Pharmacy Sciences, Federal University of Santa Catarina, Florianópolis, Brazil

**Keywords:** continuing education, continuing pharmacy education, SWOT, public health education, management training

## Abstract

**Introduction:** To train pharmacists working in the public health system, the Brazilian Ministry of Health developed a specialization course called Pharmaceutical Service and Access to Medicine Management (PSAMM) between 2010 and 2016. The course was free of charge and used e-learning as its main approach. In the end, 2,500 pharmacists were trained. The purpose of this study was to identify and analyze the strengths, weaknesses, opportunities, and threats of an in-service and e-learning course for pharmacists working in a public health system.

**Materials and Methods:** Three workshops involving 67 participants were conducted at the conclusion of the course to analyze the perspective of the PSAMM course's faculty (tutors, regional coordinators, professors, and management committee) and students (pharmacists). Strengths, weaknesses, opportunities, and threats analysis and qualitative analysis methods were used.

**Results and Discussion:** The strength dimension had the greatest number of items. The qualitative analysis resulted in six categories: the category “E-learning in continuing education” had the most cited items. Internal elements such as in-service hands-on activities directly related to the professionals' roles, course contents, faculty, and the methods to offer the course (the mixed methods and materials) were positively assessed. Nonetheless, external elements were considered critical for the course's outcomes such as investments in the infrastructure of pharmaceutical services, access to the internet, local managers' support for continuing education and innovation implementation, practice of interprofessional collaboration, and political stability. The continuing education course in the public health system was affected by internal elements such as its project and structure as well as external elements such as the sociopolitical scenario. Continuing education investment must be accompanied by infrastructure investment and coordination of services.

## Introduction

The Unified Health System (SUS) provides access to health services for everyone in Brazil that are fully financed by public resources. The majority of the population relies on it (1). The right of access to medicines is ensured by ubiquitous public health care centers. The National Pharmaceutical Policy details all the activities required to promote and manage access and rational use of medicines (2004) (2). These activities are developed by pharmacists at the municipal, state, or national governance levels. In about 90% of municipalities' health departments, pharmacists manage the selection, planning, and purchase of medicines (3). Pharmaceutical services and their management in SUS are multilevel and complex. They also represent 16% of the expenditure of the health system (4).

Despite important normative framework on SUS management being available, there are still failures in the organization of pharmaceutical services in primary care. Governance of the access to medicines and management capacity are key factors to guarantee access to medicines and comprehensive therapeutic assistance. Many authors have argued that the professional skills of the currently available workforce do not correspond with the expected profile for work in SUS, particularly related to management and leadership skills (5–8).

The Ministry of Health (MoH) identified the need for training pharmacists who are in charge of pharmaceutical policy and development of services (5–7) in the municipalities (prioritizing poor municipalities, but also middle-income municipalities) in management of health systems (focused in pharmaceutical-related issues). The MoH requested one public university to develop a national course to address the need for management training. The Pharmaceutical Services and Access to Medicines Management Course (PSAMM) was the major continuing education (CE) initiative for pharmacists in Brazil, funded by the MoH and developed by the Federal University of Santa Catarina as part of the activities of the Brazilian Unified Health System Open University—UNA-SUS (a consortium of universities created by the MoH to meet the training and education needs of the SUS). The course was offered to pharmacists working in the public health system, using e-learning as the main approach.

After course conclusion with the training of 2,500 pharmacists, the MoH requested the present study with the goal of investigating the general perceptions of stakeholders (participants fo the study), including students (pharmacists), of the development of the course and the use of e-learning for this purpose.

Distance learning approaches are essential to qualify the pharmaceutical workforce in Brazil (9). Globally, accessibility and lack of time are the main barriers identified for pharmacists to engage in continuing professional development (CPD) and CE activities (10). It is worth highlighting that CE attendance constraints for the regional healthcare workforce include cost, workplace, personal factors (11), and important constraints related to geographical access (12). Such factors affect educational outcomes and health services worldwide and particularly in Brazil because it is the largest country in South America, with 5,570 municipalities, and presents huge regional and social inequalities.

Furthermore, many authors argue the importance of evaluating the impact and effectiveness of CPD/CE actions on healthcare results (12–15). Evaluations have the aim of informing decision-making and future investments in workforce training (13, 14). Decision-makers, when deciding how to use their budgets, need to take into consideration the specificities of their scenarios. In Brazil, given its territorial extension as well as the challenges to access some remote areas, this consideration becomes especially relevant. Any project of this nature, i.e., a CE training covering all the regions of the country, implies in a high volume of investment. Only a few studies focused on the infrastructural and political issues involved in the development and implementation of large-scale courses for health professionals. The purpose of this study was to analyze internal (strengths and weaknesses) and external (opportunities and threats) factors that affected the development and implementation of the PSAMM, from the perspective of the pharmacists, managers, the course staff, and the faculty members.

## Materials and Methods

### The Case Studied

#### Environment and Infrastructure of the Course

This course was free of charge. It was offered as a blended course, mainly through e-learning, with four face-to-face meetings. Besides financing PSAAM during the period of 2010–2016, the MoH was supporting other actions, such as infrastructure and workforce expansion, to develop pharmaceutical services in diverse municipalities (16). The enrollment of potential participants for the course considered the profile of the municipalities included in the “Brazil without Misery” Plan, a social policy of the federal government. In the end, 2,500 pharmacists completed the course. They work in 1,068 of the 5,570 Brazilian municipalities, covering all 27 federal states.

Students were divided into 31 regional centers, located in 17 Brazilian states, where the evaluations and four face-to-face meetings were held. The tutors (pharmacists) were from all regions of the country and linked to the regional course centers. The course had 132 peer tutors (25 students per virtual class). A national central management committee organized regional centers operated the e-learning platform, and managed pedagogical and financial matters.

#### Curriculum Overview

The PSAMM program was prepared by 50 experts from 20 institutions from all over the country. The course material was produced in printed and online versions. The online version was designed for the Moodle platform, using audio and video resources. The PSAMM total workload was 375 h, distributed over three subject matters (Table 1) and carried out over 15 months (64 weeks). According to Brazilian legislation, e-learning courses should include face-to-face assessments and face-to-face dissertation defense or a Course Completion Work presentation (17). At the first face-to-face meeting, students received the study material in a flash drive (printable text books and videos) to facilitate their access to the content because of possible problems with the quality of the internet connection in different parts of the country (18).

**Table 1:** Curriculum of the course pharmaceutical services and access to medicines management.

**Transversal Module**	**Subject Matter**	**Modules**	**Time to Complete**
Pharmaceutical services and access to medicine course (Operative Plan elaboration)	Health policies and access to medicines	Course introduction	75 h/10 weeks
		Health policies and access to medicines	
	Access to medicines management and pharmaceutical services	Drug selection	225 h/32 weeks
		Pharmaceutical logistics	
		Dispensing medicines	
	Complementary studies	Research methodology	75 h/10 weeks
		Special topics in ethics, evaluation of health technologies, technical and legal aspects related to allopathic medicines	
		Special topics in ethics, health education, technical, and legal aspects related to homeopathic medicines	
		Special topics in ethics, health education therapeutic follow-up models	
Course completion work (about the Operative Plan development)			12 weeks

PSAMM's desired learning outcomes were to develop management competencies in pharmaceutical services, including social engagement, professional autonomy, and commitment to achieve the best results from the National Pharmaceutical Policy and the health system as a whole. It targeted pharmacists (government employees) from all over the country.

The course content included theory and practices related to health policy and laws, strategic aspects of management and leadership, disseminating the principles of participatory planning, building alliances, encouraging collaboration, and involving key actors in the diagnosis of situations, as well as in the definition and execution of actions, thereby expanding the scope of pharmacists in the planning process of health services (19).

The tutors were previously trained by the course's faculty and staff through workshops and online activities. Asynchronous activities such as texts, cartoons, video documentaries, forums, and questionnaire reports were evaluated by the tutors, based on criteria previously established by the faculty. Meetings were held between tutors and the faculty to assist in correcting activities and clarifying students' questions. Synchronous activities such as chats were available to facilitate interaction between students and tutors.

Every student needed to develop an in-service training activity called the Operative Plan. It was founded on Carlos Matus' principles of Strategic Situational Planning. Such principles are based on clearly defining representative institutional aims, explaining the problems, targeting the causes of the problems, analyzing the technical-operational management capacity, mobilizing resources, and collaborating with partners (20). The aim of the Operative Plan was to help the students develop skills to use governance and management tools and strategies in real and current situations. In many cases, the Operational Plans were used by the health centers where they were developed, achieving the outcomes defined in the planning. To finish the course, every student had to present a final manuscript of the Operative Plan development process and its results (presented in the fourth face-to-face meeting, through a public presentation and a public presentation and defense of the plan).

### Study Design and Sampling Procedure

Descriptive qualitative methods followed by an online survey were adopted for this study. A sample size of 67 stakeholders were selected to represent the different regional centers from various regions of the country. The sample included course's staff (tutors); course faculty (regional coordinators, professors, and management committee members); health manager's representatives, and students (pharmacists) enrolled in PSAMM. Three independent workshops to collect data were conducted 2 years after the end of the course, in order to allow adequate time for participants to report their own perspective on the impact of PSAMM.

#### Workshop 1

Held in the Southern Region of Brazil (50 participants), was targeted at politicians and managers, course staff, and course faculty representatives. All the staff and course faculty were invited and 42 participated. The MoH, the states' and municipal governments' councils, and pharmaceutical professional bodies were invited. They nominated their representatives and 8 stakeholders participated (all of them had information about and were engaged to some extent in the course before the workshop).We organized four groups in the workshop 1, described in Table 2. Each group worked on a topic. The participants spent 4 h in topic analysis.

**Table 2:** Description of discussion groups used at SWOT analysis: course on pharmaceutical services and access to medicines management.

**Topics (groups)**	**Topics Covered**
Curriculum	Curricular organization, management contents, content (online and PDF).
Course structure	Organization of Regional Centers, face-to-face meetings (organization, number, and programming), coordination, provision of regionalized vacancies, tutoring (selection process and training), and certification.
Communication flows	Mediation and interaction processes (students–tutors–faculty), mediation of forums, and interaction tools for students.
Learning tools	Operational Plan, tests, forums, asynchronous activities, synchronous activities, individual and group activities, and course completion work.

#### Workshops 2 and 3

Conducted in the Southeast (9 participants) and Northeast (8 participants) regions of the country, were target at PSAMM pharmacists who completed the course. These pharmacists received an invitation through e-mail and phone calls, without exclusion criteria. Those who were available and agreed to participate were included. During Workshops 2 and 3, participants discussed all four topics. Each workshop lasted 4 h.

### Data Collection Procedures

The strengths, weaknesses, opportunities, and threats (SWOT) analysis approach was used for data collection. It is a strategic management tool applied to policy diagnosis and planning as well as decision-making processes (21–23). It is also used to analyze and evaluate education strategies in health (24). It is a suitable way to identify internal (strengths and weaknesses) and external (opportunities and threats) factors affecting the object studied. It facilitates the analysis of the interrelationships of these factors and supports future decision-making (23).

To guide the procedures in the workshops held, four topics were defined in the SWOT analysis: curriculum, course organization, communication flows, and learning tools, described in Table 2. The analysis was performed in two stages. First, the participants were asked to record their perspectives on the course identifying the SWOT of the PSAMM, using cardboard cards. All cards were disposed on a wall frame and all the participants could read them. The group moderator invited participants to discuss the cards shown, arguing the importance/unimportance of each as a factor that affected the course. Then, cards were grouped according to the participants' consensus. All the discussions were audio recorded. The moderation in all workshops was performed by four researchers who did not participate in the evaluation process.

An online survey was then developed, and a link sent to all participants. The survey required respondents to share their level of agreement on relevance and importance of each item. For the relevance analysis, a 5-point Likert scale was employed (I totally agree/I partially agree/I do not agree nor disagree/I partially disagree/I totally disagree). For the importance analysis, a 3-point Likert scale was used (very important/important/unimportant). Items that scored more than 80% for both agreement and importance were subject to further analysis by the participants.

In the results session, similar items from different workshops were grouped to facilitate the discussion. When necessary, the results were rearranged amongst the dimensions to ensure compliance with the methodology (22). Participants received no financial incentive to participate in the workshops.

### Qualitative Data Analysis

The tapes of each session were transcribed into a Word® document. Three trained researchers with experience in qualitative methods independently analyzed the SWOT results (described in **Table 5**) and the transcription of the discussions that took place during the sessions through thematic analysis. The analysis focused on identifying patterned meanings and organizing analytical categories to further discuss the results.

To achieve the interpretations, we followed the script proposed by Pope, Zieblnad, and Mays (25) for deductive analysis. Each researcher proposed five to eight categories. The three researchers met as a group and resolved any differences in categorization through a process of reaching consensus. The construction of the categories started from the search for the interpretation of the results' meanings, as presented by each group of participants for each SWOT dimension, as well as the associations between them. Special attention was given to the contradictions shown between what was described as strengths and weaknesses, or opportunities and threats. Finally, an explanation was constructed based on the findings (25).

### Ethical Considerations

This study was approved by the Ethics Committee on Research with Human Beings (CAAE: 46912815.0.0000.0121). All participants were asked to provide written consent. The information was anonymized, and no personal identifiers were used during data analysis or publication.

## Results

The profiles of the participants in Workshops 1, 2, and 3 are described in Table 3. The majority of the participants were female (70%), as it is the majority of pharmacy workforce in Brazil. The sample covers all five Brazilian geographical regions. Workshop 1 identified 12 factors (three strengths, three weaknesses, three opportunities, and three threats) concerning each topic. Workshops 2 and 3 listed up to 10 factors each.

**Table 3:** Attendees' profile of the SWOT analysis workshops: course on pharmaceutical services and access to medicines management.

**Workshops**	**Category**	**Women**	**Men**	**Geographical regions**					**Total**
				**N**	**NE**	**CW**	**S**	**SE**	
Workshop 1 Total = 50	Politicians and managers (representatives of the MoH, and of State and Municipal governments)	7	1	1	4	2	0	1	8
	Professional bodies representatives	6	1	1	0	1	5	0	7
	Course staff and faculty	24	11	2	8	3	10	12	35
Workshops 2 and 3 Total = 17	Students (pharmacists)	10	7	0	9	0	0	8	17

*Brazilian Regions: N, North; NE, Northeast; CW, Center-west; SE, Southeast; S, South*.

After the agreement on relevance and importance analyses of the listed items *via* the online survey, the items that had more than 80% were included in the results (results of “agree totally/partially agree” and were “very important/important”). There were 29 items after the selection on the dimensions of strength, weakness, opportunity, and threat. Table 4 shows the number of items from the workshops. Similar items from Workshops 1, 2, and 3 were grouped to facilitate discussion. The strength dimension had the greatest number of items (10), followed by the threat dimension (7 items).

**Table 4:** Quantitative description of results from workshops 1, 2, and 3 (SWOT analysis): course on pharmaceutical services and access to medicines management.

**SWOT analysis**	**Workshop 1**		**Workshop 2/3**		**Total items held Workshop 1 + 2/3**	**Total items after grouping**
	**Items**	**After 80% Likert scale**	**Items**	**After 80% Likert scale**		
Strength	12	12	10	10	22	10
Weakness	12	6	10	4	10	6
Opportunity	12	12	7	7	19	6
Threat	12	11	8	6	17	7
Total	68	29

Six categories were identified in the qualitative analysis: management training, e-learning in continued education, the course's infrastructure, issues related to public health services' working conditions, interprofessional collaboration in practice, and the political and social context in Brazil. The results of Workshops 1, 2, and 3 are presented in Table 5 and summarized below. E-learning in continuing education (CE) was the most cited category (eight related items from the SWOT analysis).

**Table 5:** Results from workshops 1, 2, and 3 (SWOT analysis): course on pharmaceutical services and access to medicines management course.

**Item**	**SWOT dimension**	Workshops^*^	**Analytical categories**
Flexibility to study in alternative time, as it is a distance course.	Strength	2/3	E-learning in continuing education
Knowledge obtained when executing the Operative Plan with several actors involved in the workplace, such as other professionals, managers, patients.	Strength	2/3	Management training/interprofessional work
Interaction between academy and health care professionals promoted by the development of practical and theoretical activities.	Strength	1/2/3	Course organization
Operative Plan elaboration and use as active methodology, developed and applied by the students in their workplace, stimulating them to take initiatives, discuss health care needs and potential problems of daily work, seeking to establish priorities in the search for solutions together with other actors involved in pharmaceutical care.	Strength	1/2/3	Management training
Study of subjects related to management, planning, and evaluation.	Strength	2/3	Management training
Quality of the didactic material that have fostered important reflections and discussions beyond technical aspect, with socio-political contextualization.	Strength	2/3	Management training/E-learning in continuing education/course organization
Course gratuity and the importance of public funding to educational strategies for professionals, considering regional context and health service needs.	Strength	1	Course organization/political and social scenario in Brazil
Logistical and technical support well-structured, with centers in all country regions.	Strength	1	Course organization
Transversal approach of management module, allowing the expansion of the interface with other contents in pharmaceutical and social sciences.	Strength	1	Management training
Activities such as discussion forums favored learning by promoting students' integration, knowledge sharing, and networking.	Strength	1/2/3	E-learning in continuing education
Accomplishment of only four face-to-face meetings throughout the course.	Weakness	2/3	E-learning in continuing education /course organization
Difficulty in implementing practical activities proposed by the course in health care facilities, due to some other processes already in place in the health centers.	Weakness	2/3	Issues related to public health services' working conditions
Some academic supervisors for the course completion work did not have enough experience in public service management.	Weakness	1/2/3	Management training
Some faculty members responsible for the elaboration of didactic material had little experience in distance learning.	Weakness	1	E-learning in continuing education
Tutors' insufficient pedagogical training to mediate learning processes and the programmed practical activities, despite their technical qualification in pharmaceutical services.	Weakness	1	E-learning in continuing education
At the end of the course, official online tools for collaborative networking among students across the country were unavailable.	Weakness	1	Course organization
Political scenario favorable to expansion and enhancement of pharmaceutical services.	Opportunity	1/2/3	Political and social scenario in Brazil
Increased recruitment of pharmacists in public health care facilities.	Opportunity	2/3	Political and social scenario in Brazil
Federal government strategies to foster CE process at national level, through funding/partnerships with universities.	Opportunity	1	Political and social scenario in Brazil
Support from managers/heads allowing pharmacists to take part in the course.	Opportunity	1/2/3	Issues related to public health services working conditions
Presence of new digital information and communication technologies to train pharmacists working in SUS.	Opportunity	1	E-learning in continuing education
Existence of a collaborative network of public universities with MoH for continuing training of SUS professionals.	Opportunity	1	Political and social scenario in Brazil
Little practice of interprofessional work in health care facilities.	Threat	2/3	Interprofessional work
Limited managerial understanding in regard to the pharmacist's role in the health care facilities and the dimension of national pharmaceutical policy.	Threat	1/2/3	Issues related to public health services working conditions
Political, economic, and institutional instability in Brazilian public services and institutions.	Threat	1/2/3	Political and social scenario in Brazil
Expressive social, economic, and cultural differences among Brazilian geographic regions.	Threat	1	Political and social scenario in Brazil
Students lack of experience with communication technologies (platform, computers, etc.) and limited supply of good quality internet in different regions of the country.	Threat	1	E-learning in continuing education
Precariousness of health professionals' working conditions in the country.	Threat	1	Issues related to public health services' working conditions/political and social scenario in Brazil
Lack of municipal management support in implementing professional continuing education activities in practice.	Threat	1/2/3	Issues related to public health services' working conditions

* *Participants—-Workshop 1: Politicians and managers; Professional bodies' representatives; Course staff and faculty/Workshops 2 and 3: Students (pharmacists)*.

The main strengths identified are related to the didactic and structural organization of the course and the training focused on management. The pharmacists who took the course highlighted the richness and quality of the didactic material developed for and used in the course. These students also pointed out the support system for studies as an important factor for their success in completing the training. Students considered the hands-on activities important to the development of skills that were immediately applied to their daily practice.

Most of the weaknesses were identified by non-students (the managers, tutors, and staff professors who took part in workshop 1) and were related to their concerns about having little previous experience in the application of distance education. On the other hand, the pharmacists, who were the students in the course, highlighted some difficulties in the development of the course's practical activities; students asked for conditions to carry the activities out in the health services. Such conditions were not always present.

Factors related to the political and social scenarios dominated the opportunities identified by all participants. The period during which the course was developed was marked by investments in public health and education policies, including the hiring of pharmacists and the offering of CE. Participants stressed the importance of such investments to provide opportunities for improving access to medicines and pharmaceutical services in the country.

Issues related to work conditions in the public health services were prevalent among the identified threats, highlighting the necessary relationship between CE and the generation of material and managerial conditions for the development of health services. Participants, students and non-students, thought that the political and social scenario was also noteworthy. The time frame during which this study was carried out was marked by political changes, budgetary restrictions, and divestments in public policies. Participants perceived these conditions as threats to the continuity of CE programs like this course.

## Discussion

The study enabled us to analyze the CE program in its internal characteristics and in relation to the external scenario in which the course was offered.

### Context of PSAMM Development

Political and social scenario in Brazil; Issues related to public health services' working conditions; E-learning in continuing education.

In Brazil, pharmacists have been staffing public health services for many years. However, their inclusion in primary care health teams is relatively recent. The data indicate a 75% increase of pharmacists in the primary care workforce from 2008 to 2013 (26), which was recognized as an opportunity by the participants. About 20% of the students declared having a temporary or commissioned work contract, both without job stability, when they joined PSAMM. The instability of labor relations and contracts increased job turnover and threatened their ability to attend CE activities. Job instability has been identified as a challenge for the management of work in SUS (26, 27). During the course, some students became unemployed, a complication that hindered their accomplishment of the Operative Plan and course completion. The work conditions are also related to regional differences in the infrastructure of primary healthcare facilities that influence the capability of the pharmacists of implementing innovations and a broader scope of practice (2, 5).

Participants also emphasized that the political and economic scenario is a threat. Investment in public services has been declining, owing to the current political situation, including the 2016 political coup. The political crisis promoted a major change in public policies, with the adoption of austerity measures resulting in a reduction of investments throughout the public sector (28). In a country with such inequality, reducing public health financing has a great impact on the poorest population. The Brazilian statistics related to Covid-19 infections and deaths in 2020 is a cruel example of the consequences of the divestment in public health. There is a promise of further diminishing investments in the health sector in the coming years. Among other consequences, it suggests a reduction in CE courses and activities for health professionals, leading to even more dire circumstances.

The PSAMM was offered during a historical period that was described by all workshop participants as a favorable political scenario for the expansion and enhancement of health policy. Federal public funding for access to medicines and pharmaceutical services increased from around R$2 billion in 2003 to ~R$15 billion in 2015 (29). During this period, the Brazilian National Pharmaceutical Policy (promulgated in 2004) provided pharmaceutical services expansion by hiring more professionals, including pharmacists, to health teams (29).

Public funding is important and necessary for effective CPD/CE strategy. It was fundamental to allow the implementation of this project, when the health system itself had identified the need for management training and provided it to its professionals through UNA-SUS. Political and social stability are key issues to maintain and support public investments such as health professionals' training. However, professional training should not be an isolated activity, but an investment within a developmental service project and must be accompanied by investments in infrastructure and workforce teams. As the participants indicated, good local working conditions are fundamental to provide opportunity to foster advanced practices and achieve better results.

The existence of new forms of digital communication and information technologies for the training of professionals in the SUS, such as UNA-SUS, was understood as an opportunity. However, disparities in the quality of the internet in Brazilian regions were pointed out as a threat, as it causes accessibility problems to the course. Some research has indicated that in 2014 only 54.9% of private households in Brazil had internet access (30). Localities with limited access to the Internet and computers require specific and more forgiving learning options to ensure that self-paced e-learning opportunities that do not demand steady access to the Internet are provided (31). It is important to consider possible limitations in accessing the Internet. In addition to accessibility problems, lack of digital literacy is a challenge for the performance and satisfaction of some students (13, 31, 32).

### The Management Training

#### Management Training; Interprofessional Work

The distinguishing feature of the course in addressing the management of pharmaceutical and approaching management as a cross-content matter rather than an isolated content was considered a strength by participants. Management training is a recognized skill, as corroborated by the literature (33–36), given the nature and complexity of pharmaceutical service in a public system such as SUS. The strategy of inserting real examples and reflexive activities inside the technical and theoretical contents was cited as a hit to address the multilevel and complex nature of the subject.

In all workshops, participants identified the Operative Plan as an important strength. It provided the development of skills related to management, including leadership, autonomy, proactivity, communication, and team-work.

It also enabled pharmacists to interact with other health professionals, health care facility directors, and also with the patients. Besides the development of management skills, the Operative Plan was underlined as an adequate strategy to promoting such interactions and was supported in the health care facility and professional engagement contexts. However, one of the threats quoted was the poor experience of interprofessional collaboration in health services. Some health centers' managers and staff were not open to new ideas about service organization and management, and did not support the Operative Plan development. It can limit opportunities for developing new practices arising from the exercise of the Operative Plan.

In the literature, despite the recognized impact of interprofessional work on health care (37), many difficulties are reported in its implementation. They are attributed to the lack of knowledge of the pharmacist's role in the health care process (38). Pálsdóttir et al. (12) highlighted interprofessional training as a promising strategy and intervention, as it provides health professionals with education that is relevant to the context of the health system. A metasynthesis (39) highlighted that educational strategies which do not consider learning at the workplace or the local context have no impact on daily services.

The Operative Plan performed a didactic task and also an actual intervention in health services. It was an important method to bridge the gap between the contents studied and their application in the service. It encouraged interprofessional collaboration in the pharmaceutical system management to promote better access to medicines and their rational use. Our findings indicate the development of management skills as a result from the combination of the ongoing pedagogical processes and the circumstances of the practice scenario. The results demonstrate that theories must be studied along with practical activities in actual services. The local manager should be an articulator and facilitator of CE processes to enable the positive impact of such investments in training programs.

### The Educational Approach: Course Organization

Brazil's geographical, cultural, and social diversity can be noticed in its pharmaceutical system management, as highlighted by the participants. Accommodating this complexity of professional and service organization at municipal, state, and federal levels has posed the challenge of designing this educational strategy when planning PSAMM.

The organization as a blended program with e-learning as the main approach was an adequate strategy and considered a strength, however the number of four face-to-face meetings was mentioned as a weakness. Participants considered face-to-face meetings to be highly valuable, and they would have liked to have had more meetings. The PSAMM has succeeded in achieving the goal of training professionals from all the regions of the country, despite the vast geographical distances. This was possible due to the modality of learning chosen for the program. Many municipalities are located in remote and less privileged geographic regions in terms of CE offers. Nonetheless, face-to-face meetings have an important role in professional training—motivating participation and encouraging networking. Authors stress that blended programs with a mix of synchronous, asynchronous, co-located, and remote learning opportunities, are most likely to balance face-to-face learning benefits with e-learning flexibility (13), as corroborated by this study's results.

Internet accessibility constraints require flexibility in the course's organization to deal with any administrative or pedagogical problems and help students to complete activities and evaluations. During the course, technical issues faced by students led the central committee to post-pone scheduled activities and only asynchronous activities were mandatory. Other strategies adopted by the PSAMM program were the supply of teaching materials on flash drives and the provision of computers with internet access for students by the regional centers. It is also necessary to identify different levels of digital skills among students (32) and offer support, such as training on accessing the platform and using e-learning tools, having tutors capable of supporting students, and decentralizing support in regional centers.

The tutors' engagement was also fundamental in supporting students with low digital skills. They helped students deal with the restrictions and manage course activities. In Workshop 1, participants recognized tutors' technical qualifications in pharmaceutical services, but indicated insufficient pedagogical training for mentoring learning processes. Training tutors for distance learning activities is a well-recognized need and ought to be an important investment in developing e-learning programs. In this study, only non-alumni participants reported this concern. In Workshop 1, the little previous experience in distance education was a theme that emerged as a possible weakness. However, for the students (Workshops 2 and 3), little previous experience in e-learning of both tutors and faculty were not observed as weaknesses.

However, limited experience of Course Completion Work supervisors in public health service management was highlighted as a weakness in all three workshops. According to university rules, Course Completion Work supervisors should have at least, a master's degree. This issue limits the faculty to professionals with greater academic experience and, to some extent, those with little in-service experience in management. The shortage of faculty with expertise in health system management can be considered an important limitation to offer education in this area However, the expanded offer of leadership and management training for health professionals can strengthen the health system (33) and hopefully push health schools to educate for health system management.

### Some Final Words

Research (33) suggests that the majority of health leaders and managers in many low-income countries are promoted on account of clinical expertise only and usually are not prepared to manage complex health systems. Asa results, in-service leadership and management courses have been offered by governments, such as in Zambia, as an acceptable and effective model to improve knowledge and skills for health system management.

The PSAMM's issues were contextualized and analyzed within the real-life circumstances of the local and national contexts, as presented in the analyses on labor and learning conditions. The importance of developing management skills, interprofessional collaboration, and the political and social challenges of implementing a course of such magnitude in a context of profound disparities emerged.

Authors indicated that the implementation of e-learning activities can help to overcome the problems in professional training (40). E-learning has been considered a feasible way to enable CE of health professionals around the world (13, 41). The Internet is considered a good tool to provide CE programs because it offers practicing professionals the opportunity to continue in the workforce while engaging in an active-learning environment, allowing them to proceed at their own pace and convenience (13). In addition to these qualities which have been highlighted already, the present study revealed some important challenges that CE providers and health system managers need to overcome, in the program organization and delivery, as well as in the service and political contexts.

The perspectives from the diverse people involved in PSAMM through this SWOT analysis have indicated the key elements to CE development for professionals engaged in public health services. Some important lessons can be learned from this study. The success of CE projects for health professionals, particularly in public health systems, is affected not only by the course's internal elements such as the contents, as well as the organization and didactic methods, but also by external elements from the healthcare institutions, the health professionals work conditions and accessibility to technological resources, and the political and economic scenario. The planning of CE programs must consider all these issues. The characteristics of the health professionals, the health system's infrastructure, and the technology to which professionals have access are also key elements to consider.

Based on the results presented here, it is recommended that investments in CE are accompanied by: investments in improvements in infrastructure and coordination of health services; improvement of working conditions for professionals; investments in technologies for distance education; intensification of the interactivity between the health system and educational institutions; incentives for interprofessional collaboration. All these efforts need to be oriented toward a scenario of political and administrative stability, providing motivation and confidence for health professionals for their development and dedication to the health system.

Some limitations of the study should be noted. The limited time available in the workshops may have constrained the discussions. The students' workshops were performed in only two of the five geographic regions, but those are the two most populous regions of the country which concentrate most of the pharmacists who attended the course. The findings of this study might not lend themselves to generalization over other social, political, and economic conditions. Nonetheless, the internal and external factors that affected the course can advise policy makers, CE providers, and healthcare managers to prevent important constraints in the development of CE for health professionals in diverse scenarios.

This analysis of a CE strategy for health professionals intended to contribute to drive future investments in continuing education for public health systems, being able to guide projects in other countries.

## Data Availability Statement

The original contributions generated in the study are included in the article/supplementary materials, further inquiries can be directed to the corresponding author.

## Ethics Statement

The studies involving human participants were reviewed and approved by Ethics Committee on Research with Human Beings—Federal University of Santa Catarina. The patients/participants provided their written informed consent to participate in this study.

## Author Contributions

All authors have contributed equally to this work.

## Conflict of Interest

The authors declare that the research was conducted in the absence of any commercial or financial relationships that could be construed as a potential conflict of interest.
